# Transient TNF regulates the self-renewing capacity of stem-like label-retaining cells in sphere and skin equivalent models of melanoma

**DOI:** 10.1186/s12964-014-0052-z

**Published:** 2014-09-17

**Authors:** Pauline Ostyn, Raja El Machhour, Severine Begard, Nuria Kotecki, Jerome Vandomme, Pilar Flamenco, Pascaline Segard, Bernadette Masselot, Pierre Formstecher, Yasmine Touil, Renata Polakowska

**Affiliations:** Inserm U837 Jean-Pierre Aubert Research Center, Institut pour la Recherche sur le Cancer de Lille (IRCL), 1, Place de Verdun 59045, Lille Cedex, France; Univ Lille Nord de France, F-59000 Lille, France; CHULille, F-59000 Lille, France; SIRIC ONCOLille, Lille, France

**Keywords:** Cancer stem cells, Quiescence, Label-retaining cell, Melanoma, TNF

## Abstract

**Background:**

It is well established that inflammation promotes cancer, including melanoma, although the exact mechanisms involved are less known. In this study, we tested the hypothesis that inflammatory factors affect the cancer stem cell (CSC) compartment responsible for tumor development and relapse.

**Results:**

Using an inducible histone 2B-GFP fusion protein as a tracer of cell divisional history, we determined that tumor necrosis factor (TNF), which is a classical pro-inflammatory cytokine, enlarged the CSC pool of GFP-positive label-retaining cells (LRCs) in tumor-like melanospheres. Although these cells acquired melanoma stem cell markers, including ABCB5 and CD271, and self-renewal ability, they lost their capacity to differentiate, as evidenced by the diminished MelanA expression in melanosphere cells and the loss of pigmentation in a skin equivalent model of human melanoma. The undifferentiated cell phenotype could be reversed by LY294002, which is an inhibitor of the PI3K/AKT signaling pathway, and this reversal was accompanied by a significant reduction in CSC phenotypic markers and functional properties. Importantly, the changes induced by a transient exposure to TNF were long-lasting and observed for many generations after TNF withdrawal.

**Conclusions:**

We conclude that pro-inflammatory TNF targets the quiescent/slow-cycling melanoma SC compartment and promotes PI3K/AKT-driven expansion of melanoma SCs most likely by preventing their asymmetrical self-renewal. This TNF effect is maintained and transferred to descendants of LRC CSCs and is manifested in the absence of TNF, suggesting that a transient exposure to inflammatory factors imprints long-lasting molecular and/or cellular changes with functional consequences long after inflammatory signal suppression. Clinically, these results may translate into an inflammation-triggered accumulation of quiescent/slow-cycling CSCs and a post-inflammatory onset of an aggressive tumor.

## Background

Human malignant melanoma is an extremely aggressive and drug-resistant skin cancer with poor prognosis if detected at an invasive stage. Despite advances in melanoma research and drug development, 10-20% of clinically disease-free patients relapse 5–10 years following an initial treatment [1,2]. This phenomenon, which is known as tumor dormancy [3], has been related to the existence of therapy-resistant cells with stem-like activity [4-6]. Recent findings suggest that cancer stem cells, in response to chemotherapy, enter protective, prolonged, but reversible, quiescence [7] and remain dormant without causing any clinical manifestations until activated [8]. Once activated, cancer stem cells (CSCs) are responsible for melanoma re-initiation, tumor progression and increased tumor aggressiveness. Mechanisms that control quiescent tumor cell activation remain poorly understood; however, cellular interactions, the immediate microenvironment of various diffusible factors or immune surveillance may be responsible. The relatively well documented connection between the incidence of cancer and chronic inflammation [9,10] prompt us to study whether pro-inflammatory Tumor Necrosis Factor (TNF) is involved in the phenotypic switch of quiescent tumor cells into their active proliferative state in melanoma. This cytokine, which was discovered by Carswell et al. [11], is considered one of the major mediators of inflammation responsible for the development of many cancers [12,13], including melanoma, upon exposure to ultraviolet radiation [14]. Using an inducible H2B-GFP tracing system, we demonstrated for the first time that TNF increases the sub-population of quiescent or slow-cycling melanoma stem-like cells. This increase was associated with the increased self-renewal and sphere-forming abilities of melanoma cells *in vitro* and their tumor-like founding capacity in an *in vivo*-like model of human skin equivalents (SEs). More importantly, by a serial transplantation of SE-tumor cells using sphere-forming assays, we found that the tumor-founding cells maintain these TNF-induced properties for generations after first exposure and that this activity may be mediated by the PI3K/AKT signaling pathway.

## Results

### Detection of label-retaining melanoma cancer stem cells *in vitro*

**Figure 1:**
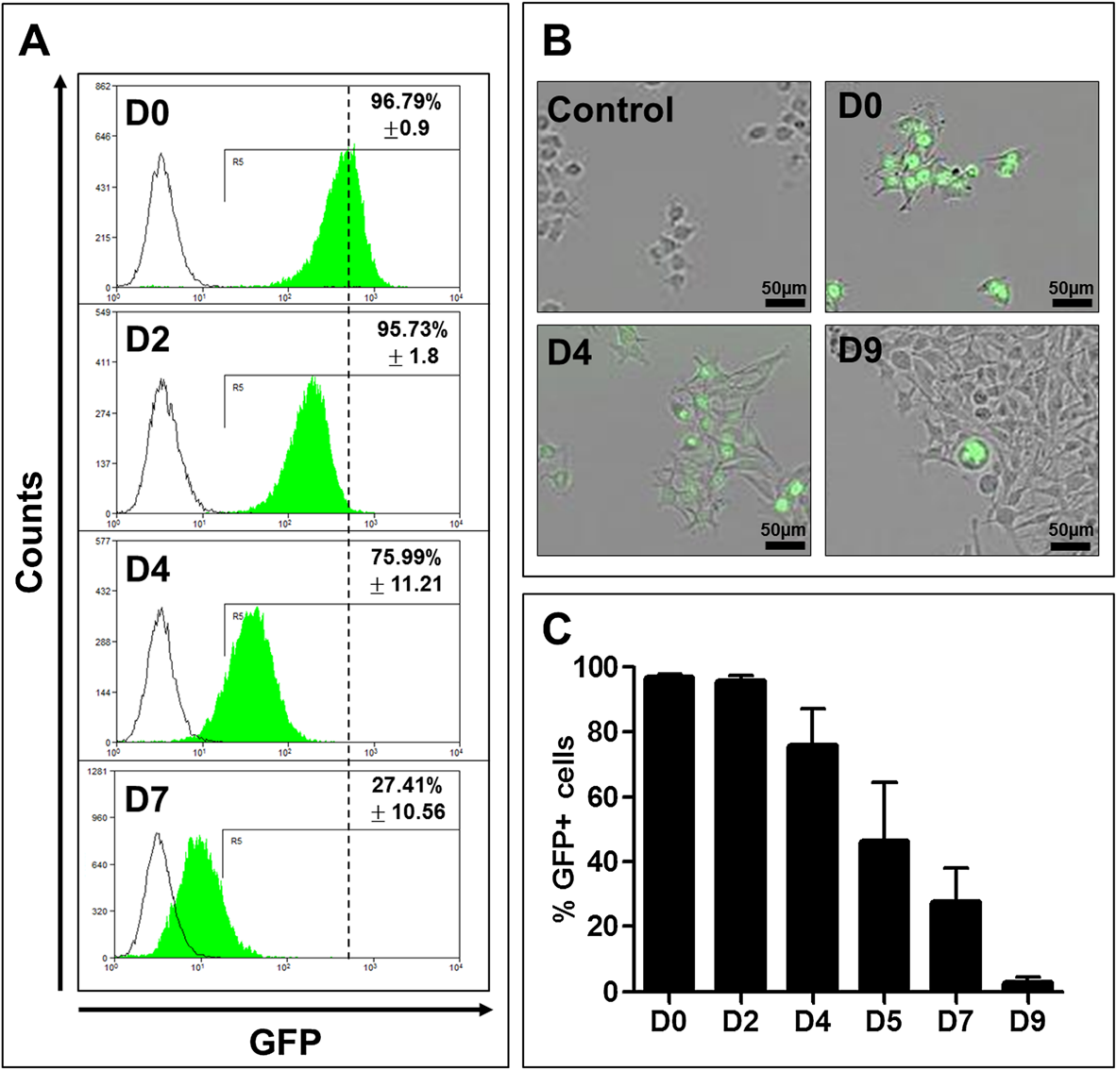
**Dividing cells with diluted Histone 2B-Green Fluorescent Protein (H2B-GFP) fusion protein monitoring cell divisional history.** HBL and SK-Mel28 melanoma cells were stably transfected with the “TET-ON” plasmid system (Materials and methods) to express inducible H2B-GFP. **A**. Flow cytometry analysis of GFP fluorescence at day (D) 0, 2, 4 and 7. GFP-negative tetracycline-uninduced cells (black lines) served as reference to gate their GFP-positive (green lines) counterparts. The numbers indicate the percent of GFP-positive cells in the total population. **B**. Representative IncuCyte images of live cell video recordings made during 9 days of culturing and illustrating a progressive dilution of GFP. Control - uninduced HBL-H2BGFP cells. Scale bar = 50 μm. **C**. Quantitative illustration of GFP dilution during 9 days of culturing.

Cancer stem cells (CSCs), similar to normal adult stem cells (SCs), remain quiescent most of the time and only infrequently enter the cell cycle to self-renew and to produce progeny committed to differentiation, composing most the tumor mass. This situation renders CSCs unable to dilute labels tracing a cell divisional history as fast as their transient amplifying (TA) progeny. Thus, these cells are recognized as the label-retaining cells (LRCs) in the tumor mass [15]. Using the *in vivo* study of Tumbar et al. [16] as a prototype, we constructed a tetracycline-inducible plasmid system expressing fused Histone B2 with Green Fluorescent Protein (H2B-GFP) and generated stably transfected clonal HBL and SK-Mel28 human melanoma cell lines (HBL-H2B-GFP and SK-Mel28-H2B-GFP, respectively). Without tetracycline, these cells were GFP-negative (Figure 1A, B), demonstrating that this system is not leaky. After 24 h of incubation with tetracycline (pulse period), 96.8% ± 0.98 of monolayer cells was labeled with GFP. A parallel flow cytometry (Figure 1A) and live cell imaging analysis (Figure 1B, C) determined that cells lost the GFP-emitted fluorescence as the cells proliferated in the tetracycline-free medium (chase period). Importantly, cell cycle progression was not affected by the H2B-GFP fusion protein ([17] and our observation). At day 9, 2.8% ± 1.8 of cells still retained their labels (Figure 1B, C); however, all cells eventually lost their labels (not shown), indicating that the monolayer culture conditions are incompatible with long-term cellular quiescence and that all cells divide, although some are slower than others.

**Figure 2:**
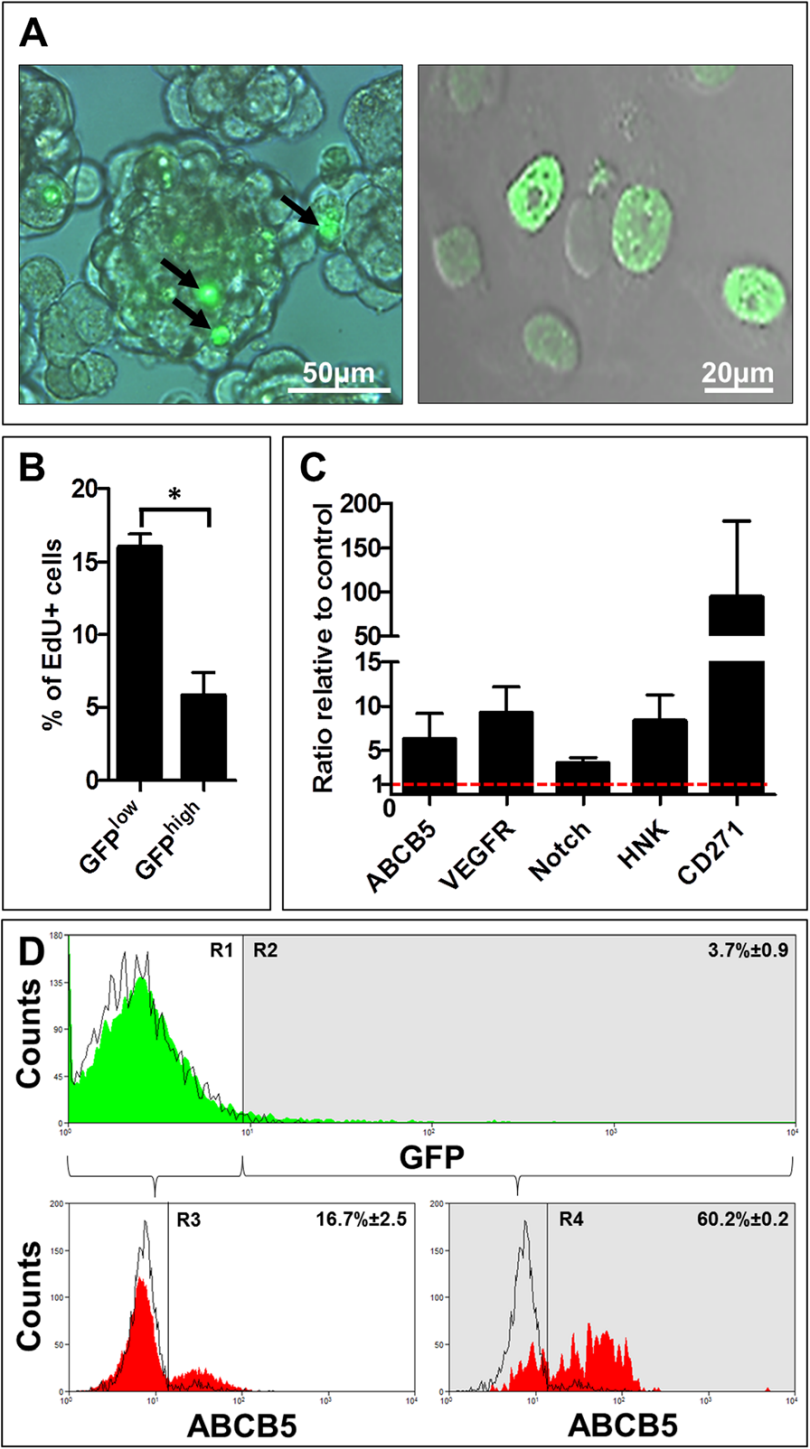
**Melanospheres contain a small subpopulation of quiescent/slow-cycling GFP** ^**high**^ **label-retaining cells (LRCs) with a melanoma stem cell phenotype. A**. Representative melanospheres (left panel, scale bar = 50 μm) formed by HBL-H2BGFP cells dividing at different rates, as reflected by differences in the GFP fluorescence intensity between dissociated melanosphere cells (right panel, scale bar = 20 μm). **B**. GFP^high^ melanosphere HBL cells cycle slower and incorporate less EdU than their GFP^low^ counterparts. **C**. These GFP^high^ cells overexpress stem cell surface markers. Histogram illustrating the ratio of expression of each marker in GFP^high^ cells to their own GFP^low^ controls, which were set at “1” and marked by the red interrupted line. **D**. Representative histograms of flow cytometry data for the surface ABCB5 marker in melanosphere HBL-H2BGFP cells. Upper histogram illustrates GFP (green) fluorescence distribution in total population. R1 is a region encompassing GFP negative (black lines) and GFP^low^ subset and R2 GFP^high^ subpopulation (shaded). Lower histograms show ABCB5 distribution between GFP^low^ and GFP^high^ (shaded) subpopulations. R3 is a boundary drawn around ABCB5^high^ cells within GFP^low^ subset and R4 within GFP^high^ subpopulation. Numbers indicate percentage of cells with a particular phenotype.

To recapitulate the more tumor-like conditions, we traced the GFP dilution in 3D sphere cultures formed by the tetracycline-induced HBL-H2B-GFP and SK-Mel 28-H2B-GFP cells. After 7 days of chase in tetracycline-free sphere-forming medium, only individual cells within melanospheres retained a high level of GFP (GFP^high^) (Figure 2A, left). Other cells fluoresced with a different intensity (Figure 2A, right), revealing heterogeneity in the proliferation rate within melanosphere cells. A double parameter flow cytometry assay evaluating a proportion of EdU-positive (EdU^+^) S-phase cells in the GFP^high^ and GFP-negative (GFP^low^) subsets of melanosphere cells established that the GFP^high^ subset contained significantly (p < 0.05) less EdU^+^ cells after 2 h of labeling than their GFP^low^ HBL-H2B-GFP counterparts (Figure 2B). Together with the above observations, an analogous decrease (1.8-fold) in the EdU^+^GFP^high^ subset of SK-Mel28-H2B-GFP demonstrates the relative replicative quiescence of GFP^high^ cells. Reversibly quiescent or slow-cycling cells were shown to have a SC phenotype [15,16,18]. A comparative flow cytometry analysis of stem cell markers with the GFP content revealed that the GFP^high^ melanosphere cell subset was enriched in cells expressing well established melanoma stem cell markers, including ABCB5 [19], CD271 (p75^NTR^), [20] and VEGFR1 [21]; a marker of neural crest stem cells, HNK1 (CD57) [22]; and Notch1, which is a common marker for many stem cell types [23] (Figure 2C). Figure 2D shows representative flow cytometry analysis for the ABCB5 marker. In summary, these data demonstrate that the pool of GFP^high^ melanosphere cells is enriched in quiescent/slow-cycling melanoma SCs that can be easily distinguished from their fast-cycling TA GFP^low^ progeny.

### Pro-inflammatory TNF increases the proportion of label-retaining melanoma stem cells

**Figure 3:**
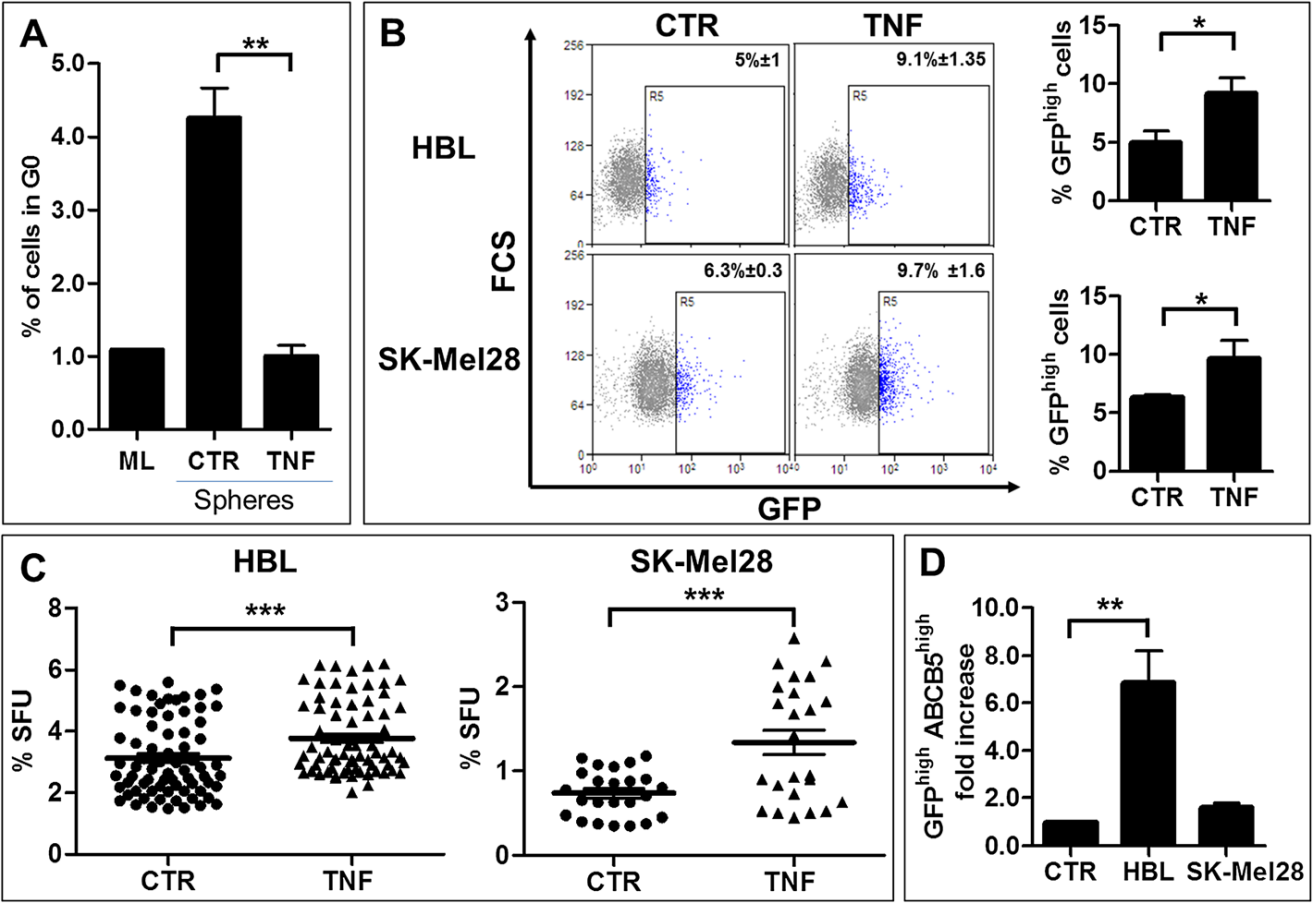
**TNF enlarges the stem-like cell compartment in human melanomas** ***in vitro*** **. A**. TNF decreased the proportion of melanosphere cells resting in the G0 phase of the cell cycle to the level found in adherent monolayer (ML) cultures. TNF (0.5 μg/ml) was added at the time of seeding cells for a sphere-forming assay. After 7 days, melanosphere cells were dissociated, reacted with an anti-Ki67 primary antibody and an anti-mouse Cy5 secondary antibody, and then stained with propidium iodide (PI) before performing the flow cytometry analysis. Ki67-negative cells in the G0/G1 fraction were considered the G0 quiescent cells. **B**. Flow cytometry of dissociated melanosphere cells revealed that TNF increased the proportion of GFP^high^ cells. Representative dot plot data and the corresponding % of GFP-positive cells (left panel) and summary histograms of all data (right panel). **C**. TNF stimulates stem cell-related sphere-forming abilities in HBL and SK-Mel28 melanoma cell lines. **D**. TNF increases expression of ABCB5, which is a melanoma stem cell surface marker, in GFP^high^ cells (GFP^high^ABCB5^high^) when compared to GFP^high^ cells in untreated controls (CTR) set at “1” for each cell line. ***p < 0.001; **p < 0.01; *p < 0.05.

Equipped with a tool that distinguishes stem from non-stem cells and knowing that chronic inflammation predisposes tissues to cancer [9,13], we aimed to determine whether inflammation affects the SC pool in melanoma, thus providing a missing link between inflammation and tumor development. The most prominent and best-characterized pro-inflammatory cytokine present in the site of inflammation is TNF [24,25], and reversible quiescence is one of the hallmarks of SCs. TNF dramatically decreased the proportion of melanosphere cells resting in the quiescent G0 phase of the cell cycle, reaching the level of adherent monolayer cultures (Figure 3A). This finding suggested that TNF stimulates the cycling of quiescent melanoma SCs. Because CSCs are defined by their LRC properties, we determined the effect of TNF on the quiescent/slow-cycling GFP^high^ subpopulation in the untreated and TNF-treated melanospheres formed by fluorescing HBL-H2B-GFP and SK-Mel28-H2B-GFP cells. The proportion of gated live GFP^high^ cells in the control HBL-H2B-GFP and SK-Mel28-H2B-GFP melanospheres amounted to 4.3% ± 1.4 and 6.2% ± 0.2, respectively, and TNF augmented their proportion to 9.2% ± 2.4 and 9.6% ± 1.5, respectively (Figure 3B). This result suggests that TNF expands the pool of LRC-GFP^high^ cells in melanospheres either by a few (not exhausting GFP fluorescence) rounds of symmetric division or by suppressing cycling of dividing melanoma SCs. When compared with the starting intensity, a general decrease in the GFP fluorescence intensity indicates that GFP^high^ cells divide, thus favoring the former possibility but not excluding the latter. Similar to normal SCs, CSCs can be recognized by their ability to proliferate as non-adherent tumor-like spheres, and a sphere-forming unit (SFU) value is an approximate indicator of the size of the SC pool [26-28]. We assessed the effect of TNF on the sphere-forming ability of HBL-H2B-GFP and SK-Mel28-H2B-GFP cells with the wild type and mutated *BRAF*^*V600E*^, respectively. Mutated BRAF^V600E^ is constitutively active in approximately 50% of human melanomas, causing their uncontrolled proliferation [29]. TNF significantly stimulated tumor-like sphere formation in both cell lines (Figure 3C), indicating that the TNF effect is BRAF^V600E^-independent and confirming that TNF expands the melanoma SC pool. As expected for melanoma SCs, live GFP^high^ cells over-expressed ABCB5 and CD271 surface markers, conferring their CSC phenotype [19,20,30], and consistently, TNF significantly (p < 0.01) increased the pool of GFP^high^ABCB5^high^ (Figure 3D) and GFP^high^CD271^high^ (2.0x ±0.2, data not shown) cells in HBL-H2B-GFP and, to a lesser extent, in SK-Mel28-H2B-GFP (1.6x ±0.2 and 1.7x ±0.6, respectively, data not shown) cell lines. Altogether, these data infer that TNF expands the pool of GFP^high^ ABCB5^high^ CD271^high^ sphere-initiating melanoma SCs. Because spheres are more tumorigenic than their adherent counterparts when grafted into severe combined immunodeficiency disease (SCID) mice [31] and because the CSC compartment is responsible for tumor development and for the severity of breast cancer [15], we presumed that TNF also predisposes to melanoma and a higher tumor burden by increasing in the CSC compartment.

### TNF inhibits melanoma cell differentiation and induces transferable changes affecting the size of the melanoma SC pool in 3D tumor-like sphere and organotypic skin models

**Figure 4:**
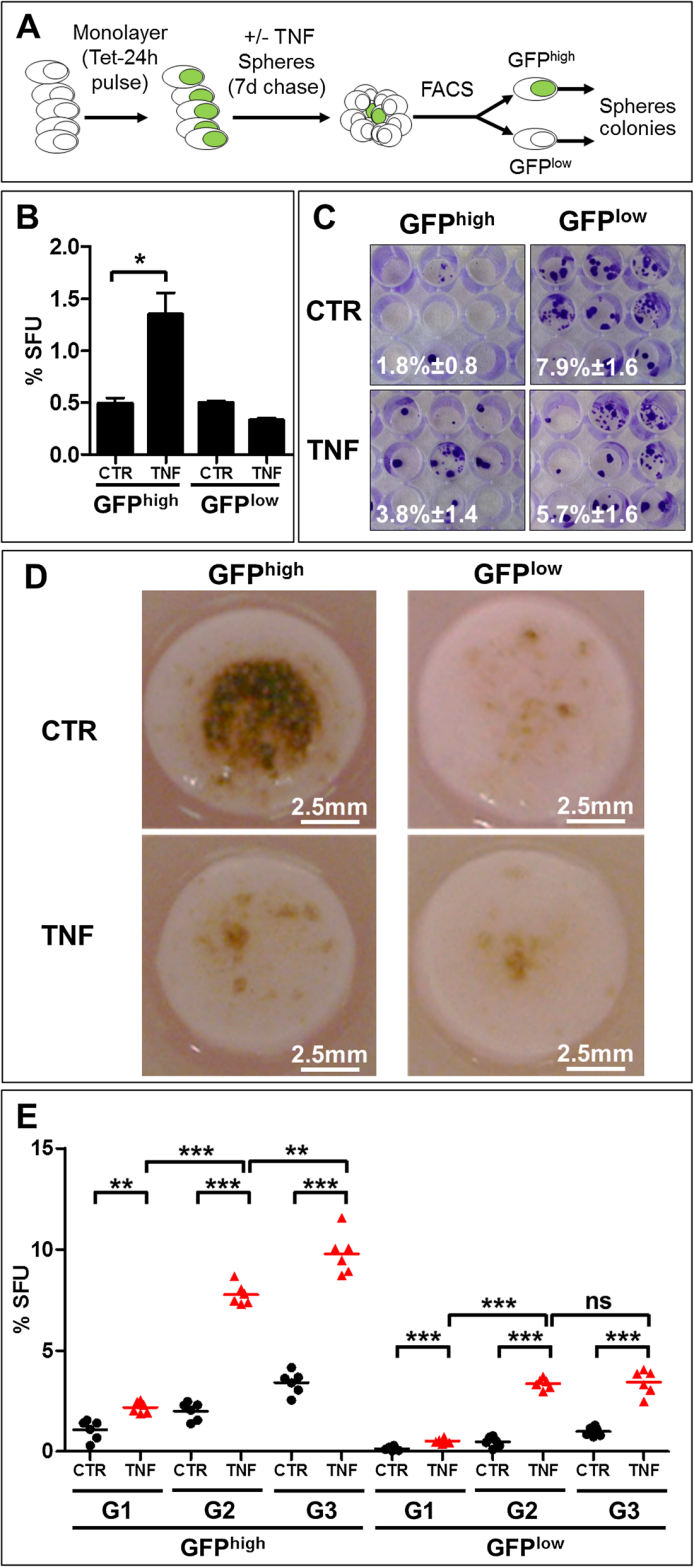
**Transient exposure to TNF induces irreversible functional changes in the GFP** ^**high**^ **stem-like cell compartment. A**. Schematic representation of the experimental design. Green nuclei refer to GFP-positive cells. **B**. TNF affects the pool of self-renewing GFP^high^ cells. Tetracycline-induced HBL-H2BGFP melanoma cells formed melanospheres in the presence or absence of TNF that 7 days later were dissociated, and cells were sorted by FACS. The GFP^high^ and GFP^low^ cells were assayed for their sphere- **(B)** and colony **(C)**-forming abilities in TNF-free medium. The histograms represent accumulated data from 24 individual samples. **C**. Representative image of clonogenic assay. The numbers indicate the % of colony-forming units. **D**. TNF blocks melanoma cell maturation. Representative skin equivalents (SEs) co-cultured with HBL melanoma cells and untreated or treated with TNF (0.5 μg/ml) for 3 weeks with TNF added to fresh medium, which was changed every 3 days. Experiments were repeated 3 times. **E**. Control (CTR-black) or TNF-treated (TNF-red) dissociated SE cells were evaluated for their ability to generate successive generations of spheres in TNF-free medium. The first (G1), second (G2) and the third (G3) generation spheres were formed during 7 days. ***p < 0.001; **p < 0.01.

To determine functional consequences of the TNF-instigated changes leading to the increase in GFP^high^ cells with the melanoma SC phenotype, we performed functional tests (Figure 4A) using tumor-like sphere cultures. Tetracycline-induced (pulse) monolayer HBL-H2B-GFP and SK-Mel28-H2B-GFP cells were used to generate spheres in the presence or absence of TNF in tetracycline-free (chase) medium for 7 days. Dissociated and sorted quiescent (GFP^high^) and TA actively cycling (GFP^low^) untreated and TNF-treated melanosphere cells were assayed for their ability to form secondary spheres and colonies in the absence of TNF. This experiment was designed to allow the identification of a TNF responding subpopulation and the estimation of the proportion of sphere-initiating CSCs in the first generation of melanospheres. The same number of sorted GFP^high^ TNF-exposed melanosphere cells produced more secondary spheres (Figure 4B) and colonies (Figure 4C) than their unexposed counterparts despite TNF absence. In contrast, exposed GFP^low^ cells formed fewer spheres and colonies then their unexposed controls. This result demonstrates 2 important events: one, that the transient exposure to TNF induces changes that persist for generations after TNF withdrawal, and two, that this long-term TNF effect is exclusively maintained by GFP^high^ CSCs. This finding suggests that TNF imprints transferable molecular changes that permanently affect the functionality of the melanoma GFP^high^ SC compartment.

To gain insight into the possible cellular mechanism(s) by which TNF maintained its effect, we recapitulated melanoma in an *in vivo*-like [32,33] skin equivalent (SE) model, which is an alternative to animal models, using sorted quiescent (GFP^high^) and fast-cycling (GFP^low^) cells in the presence or absence of systemic TNF for 3 weeks. Interestingly, control GFP^high^ HBL-H2B-GFP cells (Figure 4D) and, to a lesser extent, SK-Mel28-H2B-GFP (not shown) cells were capable of developing into the highly pigmented assembly of cells resembling melanoma tumor *in vivo*. TNF apparently limited this process in GFP^high^ SEs and had no effect on GFP^low^ SEs, which contained only a few pigmented spots. These data underline the superior tumor regeneration potential of the GFP^high^ cells over their GFP^low^ counterparts and suggests that chronic TNF either specifically eradicates the majority of GFP^high^ cells in SEs or suppresses their differentiation. The presence of some pigmented spots in all SEs independent of the condition indicates a differential cellular response to environmental clues and suggests that both GFP^high^ and GFP^low^ cell compartments are heterogeneous and that the GFP^low^ compartment contains a small subpopulation of cells with SC activity but are phenotypically undistinguishable from the non-stem GFP^low^ cells, ratifying our earlier findings [34].

To resolve whether TNF eradicates or blocks GFP^high^ cell differentiation, SE cells were recovered and assayed for their sphere-forming abilities. Apparently, GFP^high^ cells were not eradicated because these cells initiated the formation of more spheres (Figure 4E) when derived from TNF-treated SEs. Consistent with a study by Landsberg et al. [35], this result indicates that TNF inhibited the melanoma GFP^high^ cell differentiation fate. However, in Landsberg et al. study, this effect was massively reversible at the molecular level. We concluded that the TNF-induced changes targeting melanoma SC compartment are irreversible, at least at the functional level, because the TNF-reduced pigmentation was linked to a steadily increasing number of secondary and tertiary spheres formed by the sorted GFP^high^ cells dissociated from the first generation TNF-treated melanosphere in the absence of TNF (Figure 4E). A small effect of TNF on GFP^low^ and unexposed melanosphere cells confirms the existence of actively dividing CSCs [34,36] within the non-SC GFP^low^ cell subset. However, their number tended to decrease with further generations, suggesting that the GFP^low^ cells in the TNF-free environment progressively exhaust their sphere-initiation and repopulation capacity, which is apparently well preserved by the GFP^high^ cell subset that seems to attain a TNF-exposure “memory”. Interestingly, these capacities appear to be specifically restricted by the SE environment since unexposed GFP^low^ SE cells generated fewer spheres then unexposed GFP^low^ sphere cells (Figure 4B vs 4E). Collectively, all the above data demonstrated that melanoma cell lines contain a small pool of GFP^high^ABCB5^high^CD271^high^, quiescent/slow-cycling, self-renewing, melanoma stem-like cells that lose their ability to differentiate when targeted by TNF, even transiently, and that these cells appear to transfer this effect to further generations and manifest post-TNF exposure.

### Inactivation of the PI3K/AKT signaling pathway abolishes the TNF effect on the melanoma stem cell compartment

**Figure 5:**
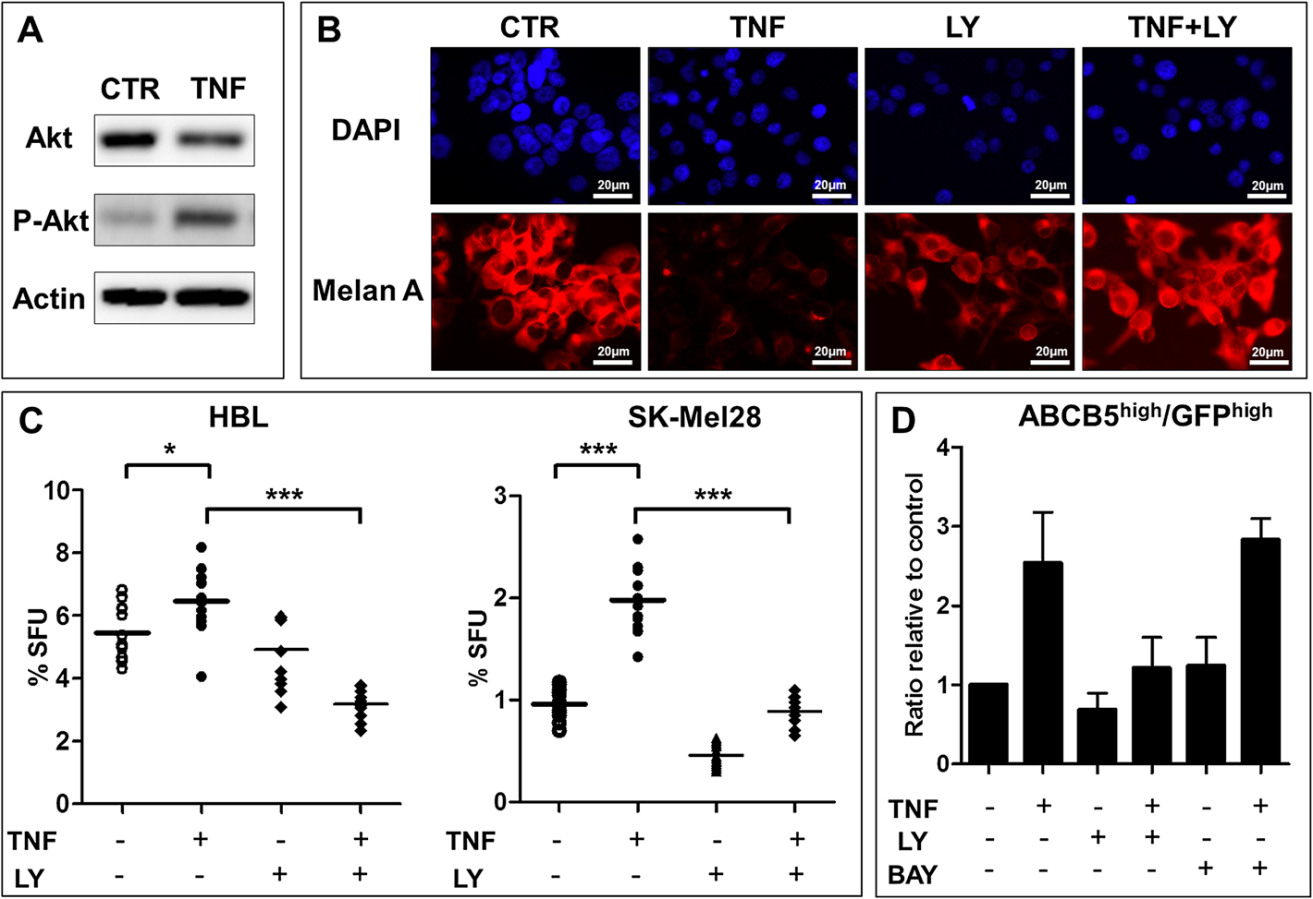
**TNF expands the subset of GFP** ^**high**^ **/ABCB5** ^**high**^ **melanoma stem-like cells through the AKT-signaling pathway. A**. Representative image of western blot (n = 2) analysis showing an increase in phosphorylated (p)-AKT in spheres formed by HBL melanoma cells cultured in the presence of TNF (0.5 μg/ml). Actin served as a loading control. **B**. Representative fluorescent microscopy images showing that LY294002 (10 μM) addition at sphere seeding in the presence or absence of TNF stimulates Melan A expression, and the morphological differentiation of melanospheres was inhibited by TNF. For each condition spheres were collected from 24 wells before dissociation and immunocytochemistry analysis. Scale bar = 20 μm. **C**. PI3K/AKT inactivation blocked the TNF-induced melanoma SC-related ability to form spheres. Melanospheres were formed in the presence or absence of TNF (0.5 μg/ml) and LY294002 (10 μM). *p < 0.05; ***p < 0.001. The data represent 2 independent experiments in 3 repetitions. **D**. Flow cytometry data showing that the PI3K/AKT inhibitor LY294002 (10 μM) added at seeding decreased the proportion of TNF-induced ABCB5^high^ cells in melanospheres formed by TNF-treated HBL cells. Note that the NFκB inhibitor BAY 11–7082 (1 μM) did not repress TNF-mediated induction. Data of at least 2 independents each combining spheres from 24 wells.

TNF-suppressed differentiation, which was accompanied by an increase in GFP^high^ABCB5^high^ sphere-initiating melanoma SCs, strongly suggests that TNF blocks the commitment of these cells to differentiation, favoring their symmetric over asymmetric self-renewal. One of the important regulators of SCs, including CSC fate, is the AKT signaling pathway [37-39]. Among its multiple functions, AKT has been shown to block the differentiation of myeloid leukemia [40] and embryonic stem cells [41]. A deregulated AKT signaling pathway is often found in melanoma [42,43], and we previously demonstrated that this pathway regulates melanoma SC quiescence [34]. Consistent with the previous findings, TNF also phosphorylated AKT in melanosphere cells (Figure 5A), and this phosphorylation was associated with the suppression of the differentiation-related MelanA expression overridden by LY294002, which is an inhibitor of the PI3K/AKT signaling pathway (Figure 5B). These data suggested that AKT might mediate the TNF-initiated inhibition of melanoma differentiation. A significant (p < 0.001) reduction in the sphere-forming capacity of melanosphere cells generated in the presence of LY204002 (Figure 5C) demonstrated that the sustained inactivation of AKT signaling reduced the melanoma SC compartment most likely by switching from symmetric to asymmetric self-renewal and by releasing the TNF-suppressed differentiation fate of melanoma SCs. Consistently, LY294002 suppressed the TNF-induced upregulation of GFP^high^ABCB5^high^ melanoma SCs (Figure 5D), strongly supporting AKT involvement in the TNF-mediated regulation of melanoma SC fate determination and functionality. Finally, because TNF-activated AKT targets NFκB, which is a well-known mediator of TNF responses [44-46] that control SC fate [47], and because NFκB can target AKT [48,49], evidencing a cross-talk between these pathways [49,50], we used the NFκB inhibitor BAY 11–7082 in combination with TNF to form melanospheres. This inhibition should distinguish which of the two pathways mediates TNF responses. As shown in Figure 5D, the NFκB inhibitor did not affect the TNF-induced upregulation of GFP^high^ABCB5^high^ melanoma SCs, demonstrating that this effect is linked to AKT rather than NFκB activation.

## Discussion

Reversible cellular quiescence is a hallmark of SCs. This ability protects these cells from a harsh environment and prevents their exhaustion imposed by constant cycling [18,51,52]. CSC entry into cellular quiescence and their post-therapeutic persistence in an apparently dormant state may be one reason why curing cancer remains difficult. Dormant cells can be activated and re-initiate tumor growth locally and in distant metastatic sites [1,53]. The exact molecular factors and cellular mechanisms governing the quiescence and activation of dormant CSCs have been intensely investigated but remain unclear, highlighting the requirement for additional research in this area. Because inflammation has been functionally related to cancer evolution and because inflammatory signals have been shown to regulate the quiescence/activation of cancer and normal SCs [8,10,47,54,55] but not much is known concerning the circuitries connecting inflammation to melanoma development, we examined the effect of TNF, which is one of the major mediators of cancer-related inflammatory responses [9], on melanoma cell quiescence and melanoma development in 3D tumor-like sphere and *in vivo*-like reconstructed skin models. Using an inducible H2B-GFP system to trace the cell divisional history *in vitro*, we identified quiescent/slow-cycling GFP^high^ label-retaining CSCs in melanoma cell lines and showed that transient and chronic TNF suppresses melanoma SC differentiation while enriching for a GFP^high^ melanosphere-initiating CSC subpopulation many generations after TNF withdrawal. This finding is consistent with a model in which inflammatory TNF signals imprint transferrable changes in the melanoma SC subpopulation, permanently affecting their fate and function and, consequentially, their progressive post-TNF expansion. Because the size of the CSC compartment is directly linked to a tumor burden [56], by enlarging this compartment, TNF may cause a predisposition to melanoma development and evolution. Our findings suggest that TNF may achieve this effect by activating the PI3K/AKT signaling pathway.

AKT signaling, which is often constitutively active in melanoma cells and in other cancer cells, regulates many cellular processes, including cell survival, metabolism and cell cycle progression [37,57,58]. Interestingly, recent findings indicate that AKT is a particularly important determinant of SC function because AKT controls SC quiescence, propagation and fate [37-40]. Recently, we demonstrated that quiescent melanoma SCs exit the G0 phase of the cell cycle in response to transient AKT inactivation; however, their cycling subset enters the quiescent state, whereas sustained AKT inhibition suppresses cell cycle progression [34]. In the present study, we found that LY294002, which is an inhibitor of PI3K/AKT, specifically blocked the TNF-driven enrichment of melanospheres in GFP^high^ label-retaining cells with the CSC phenotype and activity. This inhibition was accompanied by the acquisition of dendritic cell morphology and by the overexpression of the melanocyte differentiation marker MelanA. These data demonstrate that PI3K/AKT inactivation stimulated melanoma GFP^high^ cell differentiation, suggesting that TNF suppresses their commitment to the differentiation fate by activating AKT, consequently preventing asymmetric self-renewal and promoting symmetric self-renewal of GFP^high^ melanoma SCs. This interpretation is consistent with the observed TNF-driven increase in the proportion of GFP^high^ cells and in the sphere-forming efficiency as well as with the inhibition of GFP^high^ CSC melanogenesis in SEs and with the simultaneous acquisition of sphere-forming abilities by the SE melanoma cells. Therefore, it appears that one mechanism by which TNF and, in general, inflammation may cause cancer predisposition is a blockage of the differentiation fate in CSCs, at least partially preventing the generation of their fast-cycling destined-to-differentiate progeny. Logically, this mechanism would maintain CSCs in their primitive, quiescent/slow-cycling state and lead to their slow, but continuous, accumulation particularly because the TNF-induced changes seem to be perpetuated in the offspring of CSCs after TNF withdrawal. Currently, the mechanism of this event is unknown. Very recently Wilson et al. [59] have reported that melanoma SC maintenance is dependent on ABCB5-dependent secretion of Il1β another inflammatory cytokine. Is TNF-induced ABCB5 part of this regulatory circuit? Importantly however, the precedence of the transient induction of heritable changes after removing a stimulus has already been linked to the epithelial-to-mesenchymal transition process, creating self-renewing breast cancer stem cells from a non-stem cell population [60,61]. In another study, a “memory” of transitory FGF had a long-lasting effect on the fibroblast response to the secondary FGF stimulation; this memory reduced rather than increased their proliferation [62]. These findings underscore epigenetics and chromatin structure in controlling long-term responses, including the generation of CSCs and their phenotypic plasticity [63]. Whether similar molecular and cellular changes can be ascribed to the TNF long-lasting action remains to be investigated. However, TNF was shown to induce EMT and to create a permissive environment for a “non-CSC to CSC” conversion in breast cancer [64], and our data do not exclude the possibility that this mechanism could be responsible for the TNF-induced changes in the melanoma SC compartment. Notably, recent data provided evidence for cell competition as yet another mechanism leading to the selection and expansion of the best-fitted cell (review see [65-67]), which could theoretically also be responsible for the selection of the best-fitted melanoma SC in the TNF environment and their post-TNF expansion. Nevertheless, although the above mechanisms are possible, in the absence of evidence, these mechanisms remain yet to be proven options.

The TNF-induced differentiation repressing mechanism, which is mediated by the PI3K/AKT signaling pathway, seems to be the most probable explanation of our results and is strongly supported by the findings of other researchers. For example, TNF was shown to induce the reversible dedifferentiation of melanoma cells [35] and an increased melanocyte number while inhibiting their differentiation-related pigmentation [68]. Similarly, TNF promotes neural stem (NSC) cell proliferation but inhibits their differentiation [69] and maintains osteosarcomas in their undifferentiated state [70]. Additionally, in the hematopoietic system, TNF was shown to have a stimulatory growth effect on hematopoietic stem cells (HSCs) but to negatively regulate the growth of their more mature progenitors *in vitro* [71]. In contrast, recent findings revealed that TNF suppresses cycling HSCs and their long-term repopulating activity *in vivo* [72] and *in vitro* [73], indicating that although the TNF pathway is a critical regulator of HSC maintenance and function (reviewed in [47]), the stimulatory and/or repressive effect of TNF will depend on cell types, the responding compartment and the cell status within each compartment. Little is known concerning the effect of TNF on the melanoma SC compartment. We [34] and others [74] have shown that this compartment is heterogeneous and, as we suggested, that this compartment is composed of CSCs in at least 3 different states: quiescent, slow-cycling and fast-cycling, which are each identified by distinct phenotypes and which each have a different mode of response to environmental changes. Roesch et al. [74] determined that the slow-cycling melanoma cells, which were identified in our study as GFP^high^ cells, have the particular ability to switch between these phenotypes by assuming a distinct epigenetic state regulated by histone demethylase. Melanoma tumor growth depends on the presence of these cells. Consistently, the GFP^high^ cells in our reconstitution assay using an *in vivo*-like skin equivalent model were significantly more efficient in reproducing pigmented lesions resembling melanoma in vivo than their faster proliferating counterparts. This ability was suppressed by TNF, which simultaneously induced the number of melanosphere-inducing GFP^high^ cells. In melanospheres, these cells co-existed with their GFP^low^ progeny and persisted, although many divisions were required to form a melanosphere. This finding indicates that TNF, while blocking the differentiation fate of slow-cycling cells, reverses at least some of these cells into a quiescent state to prevent their exhaustion. These data strongly suggest that TNF maintains melanoma SCs in their primitive state and controls their plasticity. One pathway that is responsible for this effect is PI3K/AKT signaling, which regulates “stemness” in many stem cell systems [38] and was shown to be selectively inactivated in one of the symmetrically dividing cells to induce quiescence in one daughter while another continues to divide [75].

## Conclusions

In conclusion, we determined that transient TNF suppresses the PI3K/AKT-mediated melanoma SC differentiation and enlarges a GFP^high^ melanosphere-initiating CSC subpopulation that preserves the TNF-instigated changes, reinforcing their post-TNF capacity to form tumor-like melanospheres. These findings may have important clinical consequences because an acute inflammation may activate and expand pre-existing altered cells that remain clinically silent for generations until these cells emerge in a post-inflammatory environment as a primary or metastatic tumor in a more aggressive form.

## Materials and methods

### Cell line and cell culture

The human cutaneous melanoma cell line HBL was established in Professor Ghanem’s laboratory from a nodular malignant melanoma [76]. These cells were maintained in RPMI (Gibco®, Life Technologies™, France) supplemented with 10% fetal bovine serum (FBS) (Lonza, Verviers, Belgium) and 1% penicillin/streptomycin (Gibco®, Life Technologies™, France) in a humidified 5% CO_2_ incubator at 37°C. The medium was changed every 3 days. The SK-Mel28 cells were purchased from ATCC (HTB-72) and grown as recommended. Primary keratinocyte and fibroblast cultures were established using specimens of adult skin discarded after breast plastic surgery (Hôpital Roger Salengro, CHRU, Lille, France) as described previously [77]. The storage and use of human biological samples were declared and performed according to the local Person’s Protection Committee and to the ethical rules approved by the Department of Health, France. Keratinocytes were maintained in defined K-SFM (Gibco®, Life Technologies™, France) supplemented with 1% penicillin/streptomycin (Gibco®, Invitrogen™, France). Fibroblasts were cultured in RPMI supplemented with 10% fetal bovine serum (FBS) and 1% penicillin/streptomycin.

### Construction of plasmids and generation of stable transfectants expressing inducible H2B-GFP

To accomplish the tetracycline-inducible expression of fused histone 2B-green fluorescent protein (H2B-GFP), we cloned the Taq polymerase-amplified H2B-GFP gene from Addgene into the PCR8/GW/TOPO entry vector and then into the pT-Rex DEST30 destination vector using the Invitrogen Gateway cloning system and Clonase II enzyme mix. The constructed pT-Rex DEST30-H2B-GFP plasmid was used for the Lipofectamine-mediated transfection of human melanoma HBL and SK-Mel28 clones that were previously modified and preselected in the 0.5 μg/ml blasticidin-containing medium to express high levels of tetracycline-sensitive repressor (TetR) from the pcDNA 6/TR Invitrogen plasmid. HBL and SK-Mel28 cells expressing both plasmids were preselected in 400 μg/ml geneticin (G418 sulfate) and 0.5 μg/ml blasticidin and cloned using serial dilution assay. The presence of two plasmids, pT-Rex DEST30-H2B-GFP and pcDNA 6/TR, in the individual clones was verified by RT-PCR, and H2B-GFP expression was confirmed by flow cytometry. To induce H2B-GFP expression, cells were incubated for 24 h with 1 μg/ml tetracycline, which inactivated TetR and derepressed the tetracycline operator 2X TetO_2_ (Tet-ON system), permitting H2B-GFP transcription from the CMV promoter. Clones that expressed high, but not toxic, levels of H2B-GFP were chosen for further experimentation. Stably transfected cells were grown in the presence of blasticidin and geneticin to maintain TetR and H2B-GFP genes. Blasticidin was obtained from Gibco®, Life Technologies™, France, and geneticin was obtained from Santa Cruz Biotechnology. RNA extraction was performed following the manufacturer’s protocol (RNeasy Kit, Qiagen, Courtaboeuf, France). Primers were designed to amplify 305 bp cDNA fragments for the pcDNA6/TR plasmid and 187 bp cDNA fragments for the pT-Rex DEST30-H2B-GFP plasmid as follows: 1^st^ plasmid: 5′CTGGTCATCATCCTGCCTTT3′ and 5′GGCGAGTTTACGGGTTGTTA3′; 2^nd^ plasmid: 5′ACGTAAACGGCCACAAGTTG3′ and 5′AAGTCGTGCTGCTTCATGTG3′. RNA was transcribed into cDNA using random hexamers, recombinant RNasin® ribonuclease inhibitor (Promega, France) and M-MLV reverse transcriptase (Promega, France). PCR was performed using GoTaq® Flexi DNA polymerase (Promega, France).

### Generation of melanospheres

To generate primary spheres, 4 × 10^3^ cells were plated on 24-well plates coated with a 0.5 mg/ml poly-2-hydroxyethylmetacrylate (polyHEMA) ethanol solution (Sigma-Aldrich, France) to prevent cell attachment and cultured in DMEM/F12 medium (Gibco®, Life Technologies™, France), which was supplemented with 20 ng/ml EGF (Stem Cells Biotechnologies, Vancouver, BC, Canada), 1:50 B27-supplement (Gibco®, Invitrogen™, France), and 20 ng/ml rHu bFGF (PromoKine-PromoCell GmbH, Heidelberg, Germany), in a humidified 5% CO_2_ incubator at 37°C for 7 days. Tumor Necrosis Factor α (TNF) (0.5 μg/ml) from Immunotools, Germany, and/or an inhibitor of the PI3K/AKT signaling, 10 μM LY294002, or an inhibitor of NFκB signaling, 1 μM BAY 11–7082, which were both obtained from Calbiochem, France, were added at the time of sphere seeding and not re-added during the 7 days of sphere formation. Spheres were dissociated by a brief incubation with trypsin/EDTA solution (Gibco®, Life Technologies™, France) and then used as a single cell suspension for all the experiments. Cell viability was evaluated using the 3-(4,5-dimethylthiazol-2-yl)-2,5-diphenyltetrazolium bromide (MTT) assay (Sigma-Aldrich, France). After complete solubilization, the presence of blue formazan was evaluated spectrophotometrically by measuring the absorbance at 562 nm. For the LRC-assay, cells were treated with tetracycline (1 μg/ml) for 24 hours at 37°C in adherent culture and plated as above. Spheres (larger than ~50 cells) were counted under the microscope, and sphere-forming units (SFUs) (%) were estimated according to the formula: number of spheres/number of plated live cells × 100.

### Flow cytometry

Spheres containing GFP^high^ cells were dissociated, and single cell suspensions (2 × 10^5^ cells/500 μl) of HBL or SK-Mel28 cells were incubated for 45 min on ice in 100 μl of RPMI medium with the following primary antibodies: anti-ABCB5 (Rockland, Tebu-Bio, France), which was used at a 1:215 dilution, and anti-VEGFR1 (Abcam), anti-Notch (Santa Cruz Biotech), and anti-CD57 (HNK-1) (gift from Dr E. Dupin, Vision Institute, Paris or from Santa Cruz Biotech), which were used at a 1:50 dilution. After incubation, the cells were rinsed with RPMI, centrifuged and resuspended in 100 μl of RPMI with a secondary antibody, Cy5® goat anti-rabbit IgG (H + L) or goat anti-mouse (Molecular Probes®, Life Technology™, France), which was used at a 1:2000 dilution for 30 min on ice in dark. After incubation, the cells were rinsed with RPMI, centrifuged, resuspended in 500 μl of RPMI, and placed on ice before being analyzed by flow cytometry. Propidium iodide (PI)-positive dead cells were gated out and excluded from the analysis. Acquisition was performed on an EPICS-CYAN flow cytometer (Beckman Coulter France S.A.S.) and analyzed using Summit 4.3 software. GFP fluorescence intensities were recorded on the FL1 channel. Quadrants were determined based on negative control staining with a corresponding isotype antibody. *FACS sorting:* Cell sorting was performed on an FACS-ALTRA sorter (Beckman Coulter France S.A.S.). Spheres were dissociated, and the single cell suspension was adjusted to a concentration of 10^6^ cells/ml in RPMI. After excluding cell debris, the collection gates were set according to the negative (GFP^low^) control containing cells untreated with tetracycline. Cells with positive fluorescence constituted the GFP^high^ cell subset. The collected cells were centrifuged, rinsed and re-plated for sphere and colony generation.

### Generation of human skin equivalents

Skin equivalents (SEs) were prepared as described previously [78]. Briefly, dermal equivalents (DEs) were prepared using primary human fibroblasts (3 × 10^5^/DE) in RPMI with heat-deactivated fetal bovine serum (FBS), 1 mM NAOH and collagen I (rat tail collagen type I, BD Biosciences, France), which were placed into 6-well plates and allowed to contract for several days until their radii reached 5 mm. To regenerate melanoma cells in a reconstructed epidermis, a mixture of primary keratinocytes (1 × 10^5^/SE) and 1 × 10^4^/SE human melanoma cells with or without sorting were seeded onto DEs and cultured for 7 days in DMEM (Gibco®, Life Technologies™, France) supplemented with 10% FBS in the presence or absence of 0.5 μg/ml TNF. Then, the cultures were lifted at the air-liquid interface to stimulate keratinocyte differentiation. The medium, which was supplemented with 0.5 μg/ml hydrocortisone, was changed every 2–3 days. After 21–23 days of culture, skin equivalents were dissociated using 4 mg/ml collagenase for 30 min at 37°C and trypsin/EDTA for an additional 5 min. Then, melanoma cells were counted and plated on 24-well plates coated with polyHEMA in DMEM/F12 for sphere formation.

### EdU incorporation by melanosphere cells

An Alexa Fluor® 647 Click-iT Edu Flow Cytometry Assay Kit (Life Technologies) and its accompanied recommendations were used to estimate the proportion of GFP-positive and -negative melanosphere cells in S phase. Non-cytotoxic 10 μM EdU (5-ethynyl-2′-desoxyuridine) was added for 2 hours to melanosphere cultures before harvesting. Melanospheres were dissociated with trypsin/EDTA, and a suspension of 1 × 10^6^ individual cells was centrifuged, washed twice with PBS/1% BSA buffer and fixed in 4% paraformaldehyde solution for 15 minutes at RT in the dark. The fixed cells were permeabilized with saponin solution and incubated with Alexa Fluor® 7 azide in the supplied buffer for 30 minutes in the dark. Labeled cells were analyzed by flow cytometry. For the detection of EdU with Alexa Fluor® 647 azide, we used 633/635 nm excitation with a red emission filter (660/620 nm). The proportion of melanoma cells that were both EdU-positive and GFP-positive or GFP-negative was estimated by a flow cytometry quadrant analysis determining the percentage of each subpopulation. Negative control was gated using EdU unstained and tetracycline uninduced cells.

### Immunocytochemistry

HBL-H2BGFP melanospheres were cultured for 7 days in the presence or absence of TNF (0.5 μg/ml) and/or LY294002 (10 μM), which were both added at seeding. The dissociated melanosphere cells were plated on Lab-Tek chamber glass coverslips (Millicell EZ SLIDE 8-well glass, sterile Merck Milipore, Darmstadt, Germany) at a density of 15 000 cells per well. After 24 hours, the adherent cells were fixed in PAF solution, and immunocytochemistry was performed according to the standard procedure. A monoclonal anti-Melan A antibody, which was purchased from Santa Cruz Biotech, was used at a dilution of 1:100, and positive cells were detected with a secondary AlexaFluor 594 goat anti-mouse (Life Technologies), which was used at a dilution of 1:2000. Negative controls were performed by replacing the primary antibody with an irrelevant isotype. Nuclei were counterstained with DAPI. All slides were mounted under a coverslip with Vectashield mounting medium (Vector Laboratories, Nanterre, France) and were photographed using a Leica DMRB LAS3.7 fluorescence microscope.

### Western blot analysis

Western blot analysis was performed using ready-to-use NuPAGE 4%–12% Bis–Tris polyacrylamide gels according to the supplier’s instructions (Invitrogen™, St. Aubin, Paris, France). Blots were probed with the primary antibodies against Actin (Sigma-Aldrich, St. Quentin Fallavier, France), Akt and pAkt (Cell Signaling, France), followed by a horseradish peroxidase-conjugated secondary antibody (Bio-Rad, Marne-la-Coquette, France). Corresponding isotypes were used as controls. Immunodetection was performed using an ECL + chemiluminescence kit from Amersham. The band intensities in immunoblotting were analyzed and quantified using ImageJ and user-supplied algorithms.

### Statistical analysis

The results are expressed as the mean ± standard error of the mean (SEM) of at least 3 independent experiments each combining spheres from 24 wells unless indicated otherwise. A comparison between means was performed using Student’s t-test for unpaired data. When unequal variance was observed, Welch’s correction was applied. A comparison between several groups was performed using a one-way analysis of variance, followed by Dunnett’s multiple comparison test, using an appropriate control group as the reference. The statistical analyses were performed using GraphPad Prism 4.0 software. A p value of < 0.05 was considered significant.
